# Life satisfaction across sports disciplines and sports categories among Norwegian adolescents: comparisons to national data

**DOI:** 10.3389/fpsyg.2025.1577326

**Published:** 2025-06-06

**Authors:** Erik Grasaas, Øyvind Sandbakk

**Affiliations:** ^1^Teacher Education Unit, University in Agder, Kristiansand, Norway; ^2^School of Sport Science, UiT The Artic University of Norway, Tromsø, Norway

**Keywords:** adolescents, physical activity, sports, public health, health outcomes

## Abstract

**Background:**

Adolescence is a crucial time for engagement in physical activity (PA) as it linked to higher life satisfaction (LS) and future health. However, adolescents’ LS within the specific sport disciplines remains unexplored. The objective of this paper was to (1) describe LS, training behaviors and pain across 18 sports disciplines among Norwegian adolescents stratified by gender and (2) compare LS between the different sports disciplines and sports categories with nationwide data.

**Methods:**

Cross-sectional data was derived from the Norwegian Youngdata Survey, collected with nationwide representation, aggregated from 2021 to 2023. A total of 26.171 adolescents aged 16 to 19 years were included, derived from 18 sports disciplines (e.g., football, handball, etc.) and grouped into categories (e.g., endurance sports, team sports, etc.). Comparisons to national data (N = 109.469) were conducted using summary data t-tests.

**Results:**

Adolescents participating in cross-country (XC) skiing revealed the highest school satisfaction, highest PA levels, lowest pain and lowest painkiller usage across sport disciplines. Fifteen out of 18 sports disciplines revealed higher LS compared to national data, with substantial association in motorsport, XC skiing, football and handball (all, *p* < 0.002). All sport categories revealed higher LS compared to national data (all, *p* < 0.01), whereas the strongest associations were unveiled among girls (all, *p* < 0.002).

**Conclusion:**

Participation in sports, regardless of category, is associated with increased LS of Norwegian adolescents, with strongest associations unveiled among girls. With this research, we add further insights into adolescents’ life satisfaction, training, pain and painkiller use, providing novel sport-specific knowledge of both healthy and risk-behavior.

## 1 Introduction

Adolescence is a period encompassing physical, psychological, and social development, wherein the adolescents encounter several health-related behavioral choices (Fuligni, 2019; Dahl, 2004; Kumar et al., 2015). In this period, lifestyle habits are often established, and the decision to participate in organized sports and engage in physical activity (PA) emerges as a precursor to a healthy future (Kumar et al., 2015; van Sluijs et al., 2021). According to a prospective cohort study from Sweden, including 1.3 million participants, aerobic fitness in adolescence results in healthier adulthood and a longer lifespan (Högström et al., 2016). Previous research evidence has unveiled that 70% of premature deaths in adulthood have roots commencing from health-related behaviors in adolescence (Resnick et al., 2012), which makes the transition from childhood to adulthood a critical time of investment in PA considering the public health perspective.

PA in adolescence is a key factor for adolescents’ well-being and life satisfaction (LS) (Wu et al., 2017), as it is associated with enhancement in physical (Kriemler et al., 2011; Poitras et al., 2016), psychological (Biddle et al., 2011; Mahoney et al., 2006; Booth et al., 2023) and social outcomes (Burdette and Whitaker, 2005; Mendonça et al., 2014). LS is a measure of subjective well-being, which reflects all dimensions of life comprised into an overall assessment of one’s LS (Diener, 1984). Moreover, as LS tend to decrease during adolescence (Goldbeck et al., 2007), the measure is highly relevant due to comparable scoring across datasets, times, countries and ages (OECD, 2023). According to Ferguson et al. (2011), LS and school satisfaction are related among adolescents; however, there may be distinct differences, making both measures relevant to address. So far, several studies have reported a positive relationship between PA and LS in adolescence (Villafaina et al., 2021; Buecker et al., 2021; Rodriguez-Ayllon et al., 2019), including a recent Norwegian study revealing that the highest levels of PA had the strongest positive association with LS (Grasaas et al., 2024). While previous research has established that higher LS is connected to higher weekly PA, this association seems to reach a plateau (Mutz et al., 2021). Therefore, a non-linear relationship is suggested, wherein general life, leisure and health satisfaction tend to increase by each hour of leisure time sports activities, up to about 12 to 13 h per week and then gradually tend to leveling off or decrease (Mutz et al., 2021). This indicates that increased level of PA is generally positive, but that future studies should explore if the high training loads in athletes participating in sports would have some detrimental effects.

Despite the wide range of benefits, research evidence shows that PA declines during adolescence and that about 8 out of 10 adolescents worldwide do not adhere to the PA guidelines of at least 60 min per day of moderate-to-vigorous intensity aerobic activities (Guthold et al., 2020; Dumith et al., 2011). The decline of PA during adolescence aligns with findings from Norway (Grasaas and Sandbakk, 2024; Dalene et al., 2022). In addition, a recent report form the Norwegian Social Research (NOVA) reveals that approximately half of those who were active in organized sports at the start of their teenage years had quit by the age of 17, with the highest attrition rate revealed among gymnastics (73%) and the lowest attrition among adolescent participating in golf (18%) (Strandbu, 2023). Torstveit et al. (2018) revealed that participation in organized sports is associated with decreased likelihood of unhealthy lifestyle habits among Norwegian adolescents. According to Guddal et al. (2019), participation in team sports have several positive impacts on mental health among Norwegian adolescents and should therefore be encouraged.

While there are some standalone studies reporting negative health outcomes related to organized sports, such as increased risk of substance and alcohol use (Guèvremont et al., 2014; Wichstrøm and Wichstrøm, 2009), there is extensive evidence pointing to advantageous relations between participation of organized sports and positive health-related behaviors in adolescence (Kwan et al., 2014; Eime et al., 2013). However, the most physical active adolescent has revealed higher risks of acute injuries (Karchynskaya et al., 2022), yet inactive adolescents tend to have higher injury rate than the rest of the population (Costa et al., 2017). Regarding non-acute pain and the relation to PA, the research literature shows divided evidence, as previous studies have reported an increased risk (Auvinen et al., 2008; Kovacs et al., 2003; Paananen et al., 2010), a reduced risk (Guddal et al., 2019; Wedderkopp et al., 2009; Tremblay et al., 2011) and no apparent association (Aartun et al., 2016; Mogensen et al., 2007; Wedderkopp et al., 2003). However, due to the extensive body of research demonstrating strong associations between pain and well-being among Norwegian adolescents (Grasaas et al., 2020; Mikkelsen et al., 2021; Haraldstad et al., 2011; Haraldstad et al., 2017), it is highly relevant to explore pain and its management (including use of painkillers) in relation to different sport disciplines. While pain is a complex and subjective phenomenon, often conceptualized through the biopsychosocial model (Andrasik et al., 2005), its description across sports is particularly interesting given the varying physical, psychological and social demands inherent to each discipline. Hence, more research into pain experience within sports disciplines is needed. In addition, the increased use of painkillers (over-the-counter analgesics (OTCA)) among Norwegian adolescents is reported as a public health problem (Baumann-Larsen et al., 2023; Skarstein et al., 2024), and should also be assessed considering different sport disciplines.

In the systematic review by Kwan et al. (2014), they revealed that participation in organized sports reduced the risk of overall illicit drug use, particularly in higher secondary school. Furthermore, the systematic review by Eime et al. (2013) recommends that community sport participation is advocated as a form of leisure time PA for children and adolescents, in an attempt to improve not only physical health, but also promote better psychological and social well-being. In this context, girls are more likely to report lower levels of enjoyment and motivation in sports than boys, and thus more likely to drop out of sports during adolescence (Cairney et al., 2012) and girls tend to experience more pronounced health benefits by participation in sports than boys, particularly in relation to team sports caused by improved self-perception (Hopkins et al., 2022). However, participation in endurance sports may be beneficial due to lower odds of neck and shoulder pain, particularly in girls (Guddal et al., 2017). While participation in technical sports, team sports and strength/extreme sports have shown increased odds of lower back pain, lower extremity pain and pain in general (Guddal et al., 2017).

The impact of organized sports has rightfully received considerable attention in research in the last decades. Interestingly, evidence suggests that sport and PA programs can be essential for social and personal development, and the fact that providing an activity in itself may be more important for adolescents well-being than the type of activity provided (Morris et al., 2003). Hence, there is a need to further explore sports disciplines (e.g., football, handball, etc.) and sports categories (e.g., endurance sports, team sports, etc.) and their impact on adolescents’ LS.

This paper extends upon previous research by directly reporting LS, training behaviors and pain across 18 sports disciplines stratified by gender, and by comparing LS within sports disciplines and sports categories with national representative data. By identifying LS, training behavior and pain in standalone sports, new insights will potentially foster knowledge of sports specific benefits across genders and novel insight into future preventive risk assessments. Therefore, the objective of this current paper was two folded: (1) to describe LS, training behaviors and pain across various sports among Norwegian adolescents stratified by gender and (2) to compare the LS of adolescents participating in specific sports disciplines and sports categories to the general adolescent population in Norway.

## 2 Materials and methods

### 2.1 Study design and participants

This study aggregated cross-sectional data from the Norwegian Youngdata Survey, conducted in 2021, 2022 and 2023. Youngdata is an annual nationwide survey, which started in 2010. Within a span of every 3 years, close to all Norwegian municipalities has participated in the survey. The Youngdata survey represents an extensive source of nationwide information on Norwegian adolescents’ health and lifestyle (Bakken et al., 2016).

The study includes Norwegian adolescents from upper secondary school (aged 16 to 19 years of age). A total of 26.171 adolescents from upper secondary school reported participating in organized sports and were included in this current study. Responders derived from the following 18 different sports: 1. Football, 2. Handball, 3. Basketball, 4. Volleyball, 5. Bandy, 6. Ice-Hockey, 7. Cross-country (XC) skiing, 8. Alpine skiing, 9. Athletics, 10. Swimming, 11. Gymnastics, 12. Dancing, 13. Cheerleading, 14. Tennis, 15. Martial art, 16. Horse-riding, 17. Climbing, 18. Motorsport. (Bandy is a team sport similar to ice hockey, played with a small ball instead of a puck. Athletics is a collection of sporting events that includes, among others, sprints, hurdles, high jump, and javelin throw.)

The respective sports disciplines were grouped into sports categories to align with the paper’s objective, depicted in Table 1.

**Table 1:** Sport disciplines grouped into sport categories.

Individual sports	Team sports	Winter sports	Summer sports	Endurance sports	Strength/technical sports	Extreme sports
XC skiing Alpine skiing Athletics Swimming Gymnastics Dancing Tennis Martial art Climbing Horse-riding Motorsport	Football Handball Basketball Volleyball Bandy Ice hockey Cheerleading	XC skiing Alpine skiing Ice hockey	Football Basketball Volleyball Athletics Tennis Climbing Horse-riding	XC skiing Athletics Swimming Football Handball Basketball Volleyball Tennis Bandy Ice hockey	Alpine skiing Gymnastics Dancing Martial art Cheerleading Motorsport	Motorsport Alpine skiing Climbing Martial art

### 2.2 Outcomes

The Youngdata study comprises demographic measures and various health-related behavioral questions. Most of the Youngdata questions and statements are constructed by categorical responses, except the LS assessment. To provide a comparison across measures for the categorical responses, respective categorical variables were transformed into Percent of Maximum Possible (POMP) score (Cohen et al., 1999). According to Cohen et al. (1999), POMP scores can increase the interpretability and comparability (Table 2).

**Table 2:** Presentation of categorical variables converted into Percent of Maximum Possible (POMP) scores.

Categorical study variables	Rescaled into Percent of Maximum Possible score (POMP values)
Satisfaction in school	Totally disagree (0), disagree to some extent (33), agree to some extent (66), totally agree (100).
Physical activity level	Never (0), sometimes (20), 1–2 times a month (40), 1–2 times a week (60), 3–4 times a week (80), at least 5 times (100).
Organized training	Never (0), sometimes (20), 1–2 times a month (40), 1–2 times a week (60), 3–4 times a week (80), at least 5 times (100).
Training in the gym	Never (0), sometimes (20), 1–2 times a month (40), 1–2 times a week (60), 3–4 times a week (80), at least 5 times (100).
Independent training	Never (0), sometimes (20), 1–2 times a month (40), 1–2 times a week (60), 3–4 times a week (80), at least 5 times (100).
Headaches	Never (0), a few times (33), many times (66), daily (100)
Pain	Never (0), a few times (33), many times (66), daily (100)
Painkillers	Never (0), less than once a week (25), at least weekly (50), several times a week (75), daily (100).

#### 2.2.1 Life satisfaction

LS was assessed by the ladder of Cantril (1965) using the question: “Imagine a scale ranging from 0 to 10. The top of the scale (10) represents the best possible life for you, and the bottom (0) represents the worst possible life for you. Overall, where do you currently stand on this scale?” Higher scores indicated better LS. Satisfaction in school was assessed with the statement “*I am satisfied in school.”* The respondents could choose between four categories: *“totally agree,” “agree to some extent,” “disagree to some extent,”* or *“totally disagree.”* A single-item measure for LS has unveiled across populations satisfying validity, especially in adolescence, also when compared to multiple-item LS scale (Cheung and Lucas, 2014; Jovanović, 2016).

#### 2.2.2 Training

PA levels were assessed using the question, “*How often are you so physically active that you become short of breath or sweaty?*” Respondents could choose from six response alternatives ranging from “*never active*” to different times a week, up to “*at least 5 times a week*.” Type of training was measured with the question, “*How often do you train or compete in a sports club*?” and “*How often do you train in a gym?*.” Responders could choose from six response categories in both questions: “*never*,” “*sometimes*,” “*1–2 times a month*,” “*1–2 times a week*,” “*3–4 times a week*” or “*at least 5 times*.” The use of single-item assessments of PA have previously demonstrated substantial validity and reliability (Milton et al., 2011), and is considered essential in settings when device-based measures is not practicable (O’Halloran et al., 2020).

#### 2.2.3 Pain

Headaches were assessed with the question “*Have you experienced any of these problems (headaches) during the last month?* Responders could choose from five response alternatives: *“never,” “less than once a week,” “at least weekly,” “several times a week,”* and *“daily.”* Pain was assessed by the item “*Have you had any other physical health issues?.”* Responders could choose from the following response categories: “*no times*,” “*sometimes*,” “*several times*,” and “*daily*.” Use of painkillers (over-the-counter analgesics/OTCA) was measured with the question: “*How often have you used OTCA (such as paracet, ibux and similar) during the last month?”* The responders could choose from the following categories *“never,” “less than once a week,” “at least weekly,” “several times a week,”* and *“daily.”*

### 2.3 Data collection

The Norwegian Social Research (NOVA) at Oslo Metropolitan University in collaboration with the regional center for drug rehabilitation (KoRus) and the municipal sector’s organization (KS) administer the Youngdata study. The Youngdata survey is distributed as an electronic survey to the pupils during school hour with the respective teacher present. If adolescents do not choose to participate, they are offered schoolwork assignments by their teacher. According to Youngdata, the gathered information are valuable for planning public health efforts related to Norwegian adolescents (Bakken et al., 2016). The Youngdata study is financed through the national budget by funds from the Norwegian Directorate of Health (Bakken et al., 2016).

### 2.4 Ethical consideration

This current study is reported in accordance to the Strengthening the Reporting of Observational Studies in Epidemiology (STROBE) guidelines (Supplementary material 1) (von Elm et al., 2007). All participation in the Youngdata survey is voluntary. An informed written consent was obtained from all participating adolescents. The questions included in the Youngdata survey has been approved by the Norwegian Agency for Shared Services in Education and Research (ref. 821474), known as SIKT (Norwegian Agency for Shared Services in Education and Research (SIKT), n.d.). To access the information of the specific organized sports, a separate application was sent to The Norwegian Social Research (NOVA) at Oslo Metropolitan University. The application was approved (ref. 24-22), however due to GDPR and privacy regulations, this current study possesses no information of age, participating schools or municipalities in the dataset.

### 2.5 Statistical analyses

All statistical analyses were performed using IBM SPSS Statistics for Windows, Version 25.0 (IBM Corp., Armonk, NY, United States). Descriptive measures for continuous variables are presented as means and standard deviations (SDs). For an intuitive presentation and comparison across measures, the categorical variables were transformed into POMP scores (Cohen et al., 1999), which involve rescaling the original values of the variables so that the minimum value is set to 0 and the maximum value is set to 100. The remaining values are evenly distributed between these two endpoints. Consequently, the POMP scores can be interpreted as the percentage of the variable’s maximum possible score. T-test computed from summary data was performed in SPSS for the continuous variable LS, by entering the respective means with corresponding standard deviations and number of cases. To conservatively address the high number of pairwise comparisons across sports disciplines, a manual Bonferroni correction for pairwise comparisons was employed by dividing the alpha value (0.05) on number of comparisons (18 sports), rounded down the *p* value of 0.002. Given the relatively large sample size, with a high response rate across study variables, imputation nor bootstrapping was considered appropriate.

## 3 Results

### 3.1 Participants

A total of 26.171 Norwegian adolescents from upper secondary school reported participating in organized sports, herein 53.2% (*N* = 13,780) were boys and 46.8% were girls (*N* = 12,106). The included study variables revealed a high response rate, ranging from 86.0% to 99.6% (Table 3).

**Table 3:** Response rate in study variables.

Study variable	*N*	Response rate
Gender	25,886	98.9%
Life satisfaction	26,076	99.6%
School satisfaction	26,058	99.6%
Physical activity level	26,063	99.6%
Organized training	25,943	99.1%
Training in the gym	25,761	98.4%
Independent training	25,653	98.0%
Pain locations	23,741	90.7%
Headaches	22,495	86.0%
Painkillers	25,961	99.2%

### 3.2 Descriptive statistics

The highest total score of LS across sports was revealed among adolescents participating in motorsports [mean (SD): 7.8 (1.7)]. Boys participating in motorsports reported a LS score of 8 out of 10, whereas the highest LS among girls across sports was revealed in bandy and athletics, both being 7.5 out of 10 (Table 4). The highest school satisfaction was reported among adolescents participating in XC skiing [mean (SD) 84.1 (22.6)], and among boys in XC skiing [mean (SD) 86.1 (21.9)], whereas girls participating in athletics exhibited the highest school satisfaction [mean (SD) 86.9 (19.8)].

**Table 4:** Characteristics of adolescents’ LS and school satisfaction across sports disciplines expressed as mean/SD stratified by gender and total sample.

Organized sport	Study variables	Boys	Girls	Total sample
Football	Life satisfaction	7.6 (1.6)^***^ (*N* = 8,448)	7.2 (1.6)^***^ (*N* = 3,693)	7.5 (1.6)^***^ (*N* = 12,255)
	School satisfaction	84.2 (22.6)	83.7 (22.9)	84.0 (22.7)
Handball	Life satisfaction	7.6 (1.5)^***^ (*N* = 1,242)	7.1 (1.6)^***^ (*N* = 3,518)	7.2 (1.6)^***^ (*N* = 4,792)
	School satisfaction	84.5 (22.1)	83.2 (22.1)	83.4 (22.3)
Basketball	Life satisfaction	7.5 (1.6) (*N* = 475)	6.8 (1.6) (*N* = 151)	7.3 (1.6)^***^ (*N* = 638)
	School satisfaction	79.3 (24.9)	81.3 (23.2)	79.5 (24.7)
Volleyball	Life satisfaction	7.4 (1.7) (*N* = 498)	7.0 (1.7)^***^ (*N* = 645)	7.2 (1.6)^***^ (*N* = 1,162)
	School satisfaction	82.2 (24.4)	79.9 (25.4)	80.7 (25.1)
Bandy	Life satisfaction	7.6 (1.5) *N* = 183	7.5 (1.5)^***^ *N* = 61	7.6 (1.7) *N* = 246
	School satisfaction	83.7 (20.9)	77.5 (28.0)	82.3 (23.0)
Ice hockey	Life satisfaction	7.8 (1.5) *N* = 308	7.0 (2.1) *N* = 69	7.6 (1.6)^***^ *N* = 386
	School satisfaction	80.7 (23.9)	71.7 (29.0)	79.3 (25.0)
Cross-country skiing	Life satisfaction	7.9 (1.3)^***^ *N* = 425	7.4 (1.5)^***^ *N* = 375	7.7 (1.5)^***^ *N* = 805
	School satisfaction	86.1 (21.9)	85.5 (22.3)	85.8 (22.0)
Alpine skiing	Life satisfaction	7.7 (1.6) *N* = 122	7.4 (1.6)^***^ *N* = 65	7.6 (1.6)^***^ *N* = 189
	School satisfaction	78.8 (26.0)	82.8 (23.9)	80.4 (25.2)
Athletics	Life satisfaction	7.5 (1.6) *N* = 310	7.5 (1.4)^***^ *N* = 316	7.5 (1.5)^***^ *N* = 631
	School satisfaction	82.3 (23.5)	86.9 (19.8)	84.5 (22.1)
Swimming	Life satisfaction	7.7 (1.5) *N* = 289	6.9 (1.7) *N* = 258	7.3 (1.7)^***^ *N* = 556
	School satisfaction	81.5 (23.8)	80.1 (24.7)	80.8 (24.1)
Gymnastics	Life satisfaction	7.7 (1.5) *N* = 139	7.1 (1.7)^***^ *N* = 508	7.2 (1.7) *N* = 651
	School satisfaction	81.9 (23.0)	80.9 (23.7)	80.8 (23.9)
Dancing	Life satisfaction	6.7 (2.3) *N* = 38	7.0 (1.8)^***^ *N* = 685	6.9 (1.9) *N* = 736
	School satisfaction	77.8 (27.3)	80.6 (24.3)	80.4 (24.7)
Cheerleading	Life satisfaction	6.8 (2.7) *N* = 20	6.9 (1.8) *N* = 153	6.8 (2.0) *N* = 178
	School satisfaction	71.5 (34.8)	78.4 (25.2)	76.0 (28.6)
Tennis	Life satisfaction	7.4 (1.5) *N* = 160	7.2 (1.8)^***^ *N* = 163	7.3 (1.6) *N* = 325
	School satisfaction	81.2 (25.5)	82.4 (24.0)	81.6 (24.8)
Martial art	Life satisfaction	7.2 (1.9) *N* = 710	6.7 (2.0) *N* = 421	7.0 (2.0) *N* = 1,158
	School satisfaction	78.0 (27.3)	77.2 (26.2)	77.3 (27.2)
Climbing	Life satisfaction	7.5 (1.4) *N* = 124	6.8 (1.6) *N* = 109	7.2 (1.6) *N* = 241
	School satisfaction	81.3 (25.1)	74.0 (27.9)	77.5 (26.7)
Horse-riding	Life satisfaction	6.9 (2.0) *N* = 34	7.0 (1.8)^***^ *N* = 878	6.9 (1.8) *N* = 926
	School satisfaction	79.6 (28.9)	77.6 (25.6)	77.6 (26.0)
Motorsport	Life satisfaction	8.0 (1.6)^***^ *N* = 255	7.2 (1.7)^***^ *N* = 38	7.8 (1.7)^***^ *N* = 296
	School satisfaction	83.8 (22.7)	82.2 (21.8)	83.4 (23.0)

^***^p-value < 0.002 representing comparison of total sample, boys and girls against national data.

Boys and girls participating in XC skiing revealed the highest PA levels and highest levels of independent training (Table 5). Adolescents participating in XC skiing reported a mean score of 93.9, indicating high PA levels, as the score of 80 equals PA 4 times a week and score of 100 represents at least 5 times a week. The highest levels of organized training were reported by boys in ice hockey [mean (SD) 85.6 (21.8)] and girls in handball [mean (SD) 77.3 (19.8)] and the highest levels of training in the gym was reported by boys in cheerleading [mean (SD) 62.0 (41.0)] and girls in alpine skiing [mean (SD) 56.3 (38.9)]. Adolescents participating in alpine skiing reported most frequently going to the gym [mean (SD) 56.6 (35.8)].

**Table 5:** Characteristics of adolescents’ PA behaviors across sports disciplines expressed as mean/SD stratified by gender and total sample.

Organized sport	Study variables	Boys	Girls	Total sample
Football	Physical activity level	87.9 (17.0)	83.9 (16.4)	86.7 (17.0)
	Organized training	75.6 (25.5)	72.2 (21.1)	74.6 (21.6)
	Training in the gym	55.8 (34.4)	43.0 (32.6)	52.0 (34.4)
	Independent training	38.6 (31.1)	45.0 (26.4)	40.5 (29.9)
Handball	Physical activity level	90.8 (15.6)	86.2 (15.3)	87.4 (15.6)
	Organized training	81.1 (20.5)	77.3 (19.8)	78.3 (20.1)
	Training in the gym	60.2 (30.3)	46.7 (31.1)	50.3 (31.5)
	Independent training	36.0 (27.8)	41.6 (26.5)	40.2 (27.5)
Basketball	Physical activity level	86.0 (18.2)	82.0 (18.2)	84.9 (18.5)
	Organized training	74.9 (23.2)	76.5 (22.1)	75.2 (23.0)
	Training in the gym	53.9 (36.2)	33.1 (31.6)	49.3 (36.3)
	Independent training	37.2 (32.3)	32.8 (28.0)	36.1 (31.4)
Volleyball	Physical activity level	83.6 (18.9)	77.9 (18.1)	80.5 (18.6)
	Organized training	70.7 (23.8)	68.1 (21.1)	69.2 (22.4)
	Training in the gym	43.3 (36.3)	33.7 (33.4)	37.9 (35.1)
	Independent training	35.8 (29.3)	40.3 (27.4)	38.3 (28.3)
Bandy	Physical activity level	80.9 (19.8)	78.0 (20.2)	80.2 (19.8)
	Organized training	69.2 (24.9)	68.2 (26.9)	68.9 (25.3)
	Training in the gym	38.5 (36.9)	35.7 (32.9)	37.9 (35.8)
	Independent training	36.1 (31.6)	39.7 (26.2)	36.7 (30.4)
Ice hockey	Physical activity level	91.8 (14.7)	79.1 (19.2)	89.6 (16.3)
	Organized training	85.6 (21.8)	64.3 (27.2)	81.8 (24.1)
	Training in the gym	57.2 (37.2)	38.8 (34.1)	53.6 (37.5)
	Independent training	34.5 (33.2)	39.4 (29.3)	35.6 (32.7)
Cross-country skiing	Physical activity level	94.1 (14.3)	93.7 (12.8)	93.9 (13.6)
	Organized training	77.1 (27.6)	75.5 (27.6)	76.3 (27.6)
	Training in the gym	43.9 (30.0)	39.6 (29.8)	42.0 (30.1)
	Independent training	77.1 (26.8)	77.2 (21.6)	77.1 (24.6)
Alpine skiing	Physical activity level	83.9 (21.6)	85.5 (18.2)	84.7 (20.4)
	Organized training	61.5 (33.6)	72.5 (32.2)	65.1 (33.7)
	Training in the gym	57.5 (33.9)	56.3 (38.9)	56.6 (35.8)
	Independent training	48.4 (34.4)	59.7 (28.8)	52.9 (33.1)
Athletics	Physical activity level	89.2 (17.2)	87.2 (18.1)	88.0 (17.9)
	Organized training	70.8 (28.6)	73.5 (26.4)	72.0 (27.7)
	Training in the gym	45.9 (35.4)	37.8 (35.1)	41.6 (35.4)
	Independent training	60.7 (33.5)	57.5 (30.8)	58.9 (32.2)
Swimming	Physical activity level	88.2 (18.3)	84.7 (20.1)	86.3 (19.6)
	Organized training	77.1 (30.0)	73.1 (30.1)	75.3 (30.1)
	Training in the gym	43.8 (36.6)	40.6 (34.2)	42.1 (35.5)
	Independent training	43.4 (35.5)	51.6 (30.1)	47.2 (33.3)
Gymnastics	Physical activity level	85.4 (17.7)	80.1 (17.8)	81.3 (17.9)
	Organized training	67.8 (30.4)	63.7 (28.1)	64.5 (28.7)
	Training in the gym	40.1 (36.0)	31.5 (33.1)	33.5 (34.0)
	Independent training	35.0 (33.7)	38.2 (27.8)	37.4 (29.2)
Dancing	Physical activity level	81.1 (19.7)	79.9 (17.4)	79.8 (17.7)
	Organized training	32.8 (36.8)	27.8 (36.5)	28.2 (36.5)
	Training in the gym	50.5 (39.7)	29.5 (32.5)	30.4 (33.2)
	Independent training	45.9 (34.3)	42.3 (27.9)	42.5 (28.3)
Cheerleading	Physical activity level	75.0 (37.8)	81.1 (16.4)	80.1 (20.7)
	Organized training	83.0 (25.4)	72.8 (23.3)	74.1 (24.3)
	Training in the gym	62.0 (41.0)	32.3 (32.0)	36.5 (35.1)
	Independent training	59.0 (43.3)	35.2 (28.1)	39.1 (31.7)
Tennis	Physical activity level	81.5 (18.4)	73.5 (18.6)	77.5 (18.9)
	Organized training	65.3 (22.5)	55.6 (27.7)	60.4 (25.6)
	Training in the gym	42.4 (37.6)	33.1 (31.5)	37.5 (34.9)
	Independent training	37.6 (30.6)	44.4 (30.2)	40.9 (30.5)
Martial art	Physical activity level	82.8 (19.4)	76.5 (18.4)	80.4 (19.4)
	Organized training	45.2 (38.5)	38.3 (36.4)	42.9 (37.9)
	Training in the gym	49.7 (38.3)	32.7 (34.8)	43.4 (38.0)
	Independent training	44.6 (32.6)	43.7 (29.9)	44.3 (31.7)
Climbing	Physical activity level	80.0 (19.7)	74.1 (17.3)	77.1 (18.9)
	Organized training	45.2 (33.5)	44.1 (33.5)	45.0 (33.2)
	Training in the gym	37.2 (37.3)	24.5 (31.4)	31.4 (35.1)
	Independent training	54.1 (30.2)	56.4 (27.3)	54.8 (28.6)
Horse-riding	Physical activity level	75.9 (31.8)	77.8 (18.6)	77.5 (19.7)
	Organized training	57.6 (41.4)	55.2 (35.1)	55.3 (35.5)
	Training in the gym	40.6 (36.6)	27.7 (32.2)	28.5 (32.8)
	Independent training	53.3 (37.6)	53.5 (31.9)	53.6 (32.2)
Motorsport	Physical activity level	73.1 (23.8)	75.8 (18.1)	73.7 (23.1)
	Organized training	42.1 (34.5)	49.5 (30.8)	43.5 (34.2)
	Training in the gym	40.6 (37.5)	49.2 (36.7)	42.1 (37.6)
	Independent training	36.7 (32.9)	40.0 (32.7)	37.8 (33.3)

Boys and girls participating in XC skiing revealed lowest headache scores [total mean (SD) 36.8 (27.1)], lowest pain score [total mean (SD) 33.7 (25.9)] and lowest use of painkillers [total mean (SD) 21.9 (21.7)], presented in Table 6. Moreover, boys participating in cheerleading and girls participating in motorsport reported the highest headache scores (42.9 vs. 60.1), indicating an average headache frequency somewhere between a few times to many times a month. Adolescents participating in cheerleading exhibited the highest scores of pains [mean (SD) 55.3 (30.4)]. Boys in cheerleading and girls participating in bandy reported the highest levels of painkiller use (42.1 vs. 46.8), indicating a frequent average use (reference value 50 = at least weekly).

**Table 6:** Characteristics of adolescents’ pain and painkillers usage across sports disciplines expressed as mean/SD stratified by gender and total sample.

Organized sport	Study variables	Boys	Girls	Total sample
Football	Pain	33.1 (25.4)	44.7 (27.0)	36.8 (26.6)
	Headaches	26.8 (23.2)	43.3 (27.4)	32.0 (25.8)
	Painkillers	16.7 (19.2)	26.4 (21.8)	19.7 (20.6)
Handball	Pain	38.8 (27.4)	49.1 (27.3)	46.3 (27.7)
	Headaches	29.7 (24.3)	46.5 (28.5)	42.1 (28.4)
	Painkillers	18.5 (20.4)	29.4 (22.3)	26.5 (22.4)
Basketball	Pain	37.9 (29.2)	50.0 (27.3)	40.8 (29.2)
	Headaches	30.3 (25.3)	47.4 (27.8)	34.5 (26.9)
	Painkillers	14.6 (18.3)	25.8 (20.5)	19.2 (20.9)
Volleyball	Pain	36.3 (26.9)	48.7 (27.1)	43.3 (27.9)
	Headaches	28.9 (25.2)	45.4 (28.6)	38.3 (28.4)
	Painkillers	14.6 (18.3)	28.1 (23.3)	22.2 (22.3)
Bandy	Pain	33.8 (24.2)	53.0 (29.5)	36.7 (31.4)
	Headaches	28.6 (25.3)	45.4 (28.6)	38.9 (27.0)
	Painkillers	28.6 (25.3)	46.8 (31.2)	33.4 (27.9)
Ice hockey	Pain	38.0 (25.3)	51.7 (25.1)	40.7 (25.8)
	Headaches	34.1 (24.1)	44.6 (29.3)	36.4 (25.7)
	Painkillers	21.5 (21.4)	26.1 (23.3)	22.6 (21.9)
Cross-country skiing	Pain	28.1 (23.5)	39.7 (26.8)	33.7 (25.9)
	Headaches	20.1 (21.0)	38.1 (27.9)	28.9 (26.2)
	Painkillers	11.4 (16.8)	22.6 (19.7)	16.8 (19.3)
Alpine skiing	Pain	33.1 (28.0)	43.1 (24.8)	36.9 (27.4)
	Headaches	22.7 (22.5)	46.6 (33.4)	31.5 (29.1)
	Painkillers	16.6 (18.4)	29.6 (23.3)	20.9 (21.1)
Athletics	Pain	32.6 (26.9)	42.4 (27.4)	37.6 (27.5)
	Headaches	23.4 (23.8)	36.8 (27.1)	30.2 (26.4)
	Painkillers	13.9 (18.6)	21.9 (21.7)	18.0 (20.6)
Swimming	Pain	29.7 (25.6)	50.4 (28.1)	39.7 (28.8)
	Headaches	25.3 (24.1)	46.9 (29.4)	35.1 (28.8)
	Painkillers	14.9 (18.4)	26.9 (22.9)	21.1 (21.9)
Gymnastics	Pain	40.0 (30.7)	51.6 (27.4)	49.0 (28.4)
	Headaches	28.7 (26.6)	47.4 (28.0)	30.2 (26.4)
	Painkillers	17.2 (21.6)	29.0 (22.8)	26.4 (23.0)
Dancing	Pain	41.6 (29.5)	54.1 (28.9)	53.8 (29.1)
	Headaches	37.1 (28.4)	49.9 (27.3)	49.5 (27.6)
	Painkillers	25.7 (27.3)	30.0 (23.0)	29.7 (23.5)
Cheerleading	Pain	49.8 (40.4)	56.1 (28.4)	55.3 (30.4)
	Headaches	42.9 (36.7)	50.0 (28.1)	49.3 (29.7)
	Painkillers	42.1 (36.4)	36.2 (25.4)	37.2 (27.5)
Tennis	Pain	30.6 (24.8)	48.0 (27.5)	39.4 (27.5)
	Headaches	26.0 (25.7)	43.2 (26.7)	34.8 (27.5)
	Painkillers	10.4 (15.7)	24.7 (18.8)	17.6 (18.7)
Martial art	Pain	36.9 (28.2)	54.0 (28.5)	43.4 (29.6)
	Headaches	30.8 (24.9)	48.8 (27.9)	37.7 (27.6)
	Painkillers	14.9 (19.3)	26.0 (22.3)	19.3 (21.3)
Climbing	Pain	36.2 (28.8)	49.5 (27.4)	43.4 (29.6)
	Headaches	25.9 (24.7)	45.6 (29.6)	35.7 (29.2)
	Painkillers	11.6 (17.3)	23.4 (18.4)	17.7 (20.4)
Horse-riding	Pain	43.8 (31.5)	53.5 (27.6)	53.3 (27.9)
	Headaches	41.9 (34.8)	50.6 (28.1)	50.5 (28.5)
	Painkillers	28.7 (30.9)	30.6 (24.1)	30.6 (24.6)
Motorsport	Pain	34.5 (27.8)	62.2 (29.1)	38.2 (29.6)
	Headaches	31.3 (26.5)	60.1 (27.8)	34.7 (28.5)
	Painkillers	19.3 (23.6)	38.2 (24.5)	22.3 (25.2)

### 3.3 Comparison to national data

The comparable national Youngdata sample from 2021 to 2023 included 109,469 adolescents from upper secondary school, with LS scores of 7.0 (SD 2.0), wherein boys reported a LS score of 7.4 (SD 1.8) and girls 6.7 (SD 1.8). The national data includes the general adolescent’s population, meaning adolescents participating in sports, previously have participated in sports or are non-participants in sports.

**Figure 1:**
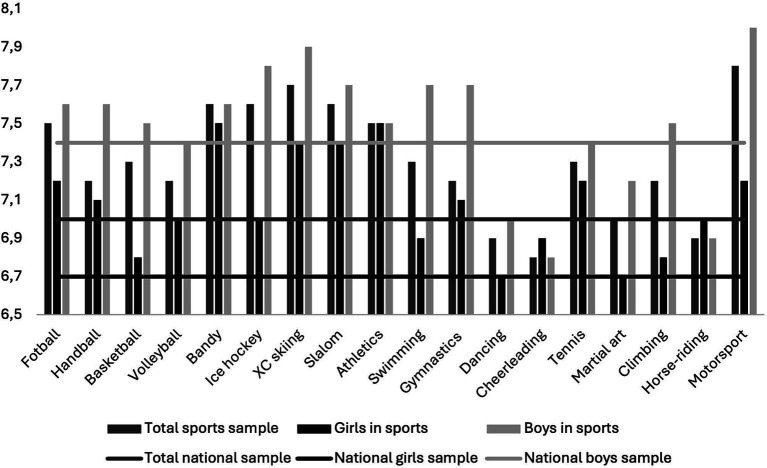
Life satisfaction scores across sports disciplines for the total sample, boys and girls compared to nationwide benchmarks (horizontal lines).

Fifteen out of eighteen sports disciplines revealed higher LS compared to the national data, with the strongest associations in motorsport, XC skiing, football and handball (all, *p* < 0.002). Findings stratified by gender revealed that girls participating in sports, regardless of discipline, exhibited at least the same or higher level of LS compared to the nationwide scores in girls (Figure 1). LS was significantly higher for girls in all sports compared to the national data (all, *p* < 0.002), except in ice hockey, swimming, climbing, martial arts, cheerleading and basketball. Boys participating in martial art, and sports predominantly played by girls, such as dancing, cheerleading, and horse-riding reported lower LS than boys from the national data.

**Figure 2:**
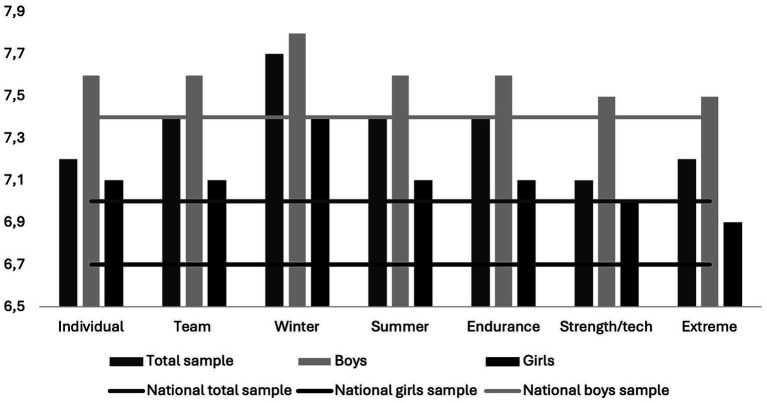
Life satisfaction scores across sports categories for the total sample, boys, and girls compared to nationwide benchmarks (horizontal lines).

Findings revealed higher LS across all sports categories compared to the national data (Table 7, all *p* < 0.01), with substantial associations unveiled among girls (all, *p* < 0.002). The highest LS scores were revealed among adolescents participating in winter sports, scoring 7.7 out of 10 and the lowest LS among adolescents participating in strength/technical sport, scoring 7.1 (Figure 2).

**Table 7:** Life satisfaction scores expressed as mean (SD) and presented by sports categories stratified by total sample, boys, and girls.

Sports categories	LS Total sample	LS Boys	LS Girls
Individual sports	7.2 (1.8)^***^	7.6 (1.7)^***^	7.1 (1.8)^***^
Team sports	7.4 (1.6)^***^	7.6 (1.6)^***^	7.1 (1.6)^***^
Winter sports	7.7 (1.5)^***^	7.8 (1.4)^***^	7.4 (1.6)^***^
Summer sports	7.4 (1.6)^***^	7.6 (1.6)^***^	7.1 (1.6)^***^
Endurance sports	7.4 (1.6)^***^	7.6 (1.6)^***^	7.1 (1.6)^***^
Strength/technical sports	7.1 (1.9)^**^	7.5 (1.8)^*^	7.0 (1.8)^***^
Extreme sports	7.2 (1.9)^***^	7.5 (1.8)	6.9 (1.9)^**^

^*^p < 0.05, ^**^p < 0.01, ^***^p < 0.002.

## 4 Discussion

This study describes novel data on LS, training behaviors and pain across sports disciplines and compared LS of sports disciplines and sports categories against the national Youngdata study. The main findings were as following: (1) Adolescents participating in XC skiing exhibited the highest school satisfaction, highest PA levels and lowest pain and painkiller usage across the sport disciplines; and (2) 15 out of 18 sports disciplines revealed higher LS and all sport categories revealed significantly higher LS compared to the national sample, with particularly strong associations among girls.

Our findings unveiled that adolescent participating in motorsports exhibited the highest LS, while adolescent participating in XC skiing exhibited the second highest LS scores, highest levels of school satisfaction, highest PA-levels, lowest pain and lowest use of painkillers, indicating healthy behavior traits. As previous studies underscore the pivotal linkage between higher PA and LS and health-related quality of life among adolescents (Grasaas et al., 2024; Villafaina et al., 2021), these coinciding findings of advantages’ behavior traits among adolescents participating in XC skiing seems logical. However, a newly published paper by Gao et al. (2024), revealed in Shandong Province unfavorable characteristics among adolescents in XC skiing, when they investigated the impact of various sports on their personality traits. These contradictory findings from Gao et al. are difficult understand, especially since their findings revealed favorable characteristic in sports with somewhat similar characteristics, such as jogging/running who also induce high PA levels via independent training. Other reasons explaining the inconsistencies in XC skiing might be linked to cultural differences and the type of population participating the sport in the two countries. Nevertheless, it seems logical that the high level of independent training in Norwegian XC skiers, and perhaps in adolescents participating in running from Shandong Province, could indicate high levels of self-discipline and determination. Possibly, behavioral traits rather than the sport discipline in itself enhance LS scores and school satisfaction, such as the higher PA-levels and independent training levels, which might be attributed to higher structure and efficiency in managing everyday tasks.

Fifteen out of eighteen sports disciplines revealed higher LS compared to the national sample. Our benchmarked national sample is comparable in terms of age, time, and country, which makes the comparison and interpretation highly interesting. Particularly, since the national sample also includes adolescents who are active in sports and those who been active in sports. The most notably findings were that girls, regardless of sport discipline, exhibited LS to be at least the same level or higher than the national sample, mostly revealed by substantial associations. The consistency in findings might be attributed to personal, peer or environmental factors on girls (Hopkins et al., 2022). Personal factors may include a higher focus on health benefits and body image among girls (Morano et al., 2020), whereas peer factors, such as peer acceptance often derives from competitive success among boys, peer relations in sports often include more emotional support among girls (Murray et al., 2022). As girls tend to struggle more with self-esteem, stress and are less physical active during adolescence, participating in sports may create both physical benefits and psychological supportive environments (Morano et al., 2020), which seems to especially crucial among girls. Although girls’ LS are lower than boys in adolescence, previous research indicates that gender differences do not extend into adulthood (Orben et al., 2022), which makes sports participation and promoting PA among adolescents’ girls a particularly critical time-window. Particularly, as research suggest that higher PA-levels have the strongest impact on girls LS (Villafaina et al., 2021).

In this study, all sport categories revealed significantly higher LS compared to the national sample. These aligns with previous research evidence by showing higher LS and favorable health outcomes among adolescents participating in sports (Torstveit et al., 2018; Guddal et al., 2019; Fernandes et al., 2024). Aligned with previous literature (Ferguson et al., 2011), the LS findings closely matched the school satisfaction scores, though not entirely, which highlights the multidimensional aspects of adolescents’ everyday life. Interestingly, participation in winter sports revealed the highest LS. These findings might be explained by the timing of data collection, as the adolescents answer the Youngdata questionnaire during the winter months. Presumably most active adolescents prefer to be in-season, rather than post-or pre-season. However, the findings of higher LS might also be explained by the continuous grinding and training outside in fresh and cold conditions, whereas the general adolescent’s population might prefer more sedentary indoor conditions. Therefore, high PA levels in winter sports might be linked to mental toughness, such as higher self-efficacy, which is suggested to be an mediator between PA levels and LS among Norwegian adolescents (Grasaas et al., 2024). Previous research has revealed robust positive associations between higher PA levels and self-efficacy across years among Norwegian adolescents (Grasaas and Sandbakk, 2024). Self-efficacy is a concept linked to positive youth development (Tsang et al., 2012) and according to the recent meta-analysis of Bruner et al. (2023), organized sports promote positive youth development through a wide range of physical and psychological benefits. This underscores the essential benefits of sport participation and higher levels of PA on adolescents’ well-being, and the importance of promoting PA to the upcoming adolescent’s population.

### 4.1 Strengths and limitations

Several key factors have contributed to the study’s rigor, validity and reliability. First, by following the STROBE guidelines, this study provides structural transparency in the reporting. The study benefits from using a benchmarked sample aggregated from a three-year period including all parts of Norway, which increases the validity and representativeness of the findings. Moreover, the high response rate and relatively high number of adolescents across sports categories and disciplines should be considered a strength. The Youngdata study have a stringent and rigor procedure of cleaning of data (Bakken et al., 2016), which identifies and exclude unserious responses (e.g., maximum scores in both LS and depression measures), thereby increases the study’s credibility.

Although data were aggregated over time, this study is using a cross-sectional design, which does not provide any causal interactions nor understanding trends over time. As the scope of the study was to compare sports disciplines and sports categories, simple summary t-tests were used, where adjusting for co-variates was not possible, which should be considered a limitation. Moreover, as pain and the use of painkillers was assessed by a recall of a month, there might be cases of recall biases. As the Youngdata includes a wide range of health-related questions, many of the single-item questions were not validated, which should be considered a major limitation. The lack of valid and reliable measures reduces the credibility of the findings. In addition, by dividing sports disciplines into categories, nuances are lost, as some sports disciplines might be considered year-round activities, which also should be considered as a limitation. Finally, future studies should include other background variables such as socioeconomic status to provide a broader understanding of this study population, including more advanced statistics, such as estimating effect sizes and adjusted regressions of different type of PA on LS within sports disciplines, across sport categories and among adolescents previously participated in sports, and among adolescents not participating in sports.

### 4.2 Perspectives

This paper extends upon previous research by reporting LS, training behaviors and pain across 18 sports disciplines and by comparing LS within sports disciplines and sports categories with national representative data. Given the insight in the specific sports disciplines, researchers, sport federation, sports clubs, schools, and trainers, should take findings of headache, pain scores and painkiller usage across gender into consideration when planning and executing preventive efforts. Regardless of sport discipline and sport category, when considering the findings in a public health perspective, we strongly advocate policy and practice to promote PA among Norwegian adolescents, as it seems to be a critical time-window of time for healthy behavior traits. The Norwegian government should invest in initiatives that prioritize making sports disciplines and PA free and accessible to all adolescents, and thereby facilitate higher levels of both organized sports and independent training.

Although this paper highlights the benefits of participating in organized sports, the overarching message to adolescents is that, regardless of the specific sport, physical activity is a crucial investment in health. Sports can be a helpful way to maintain higher physical activity levels during adolescence, but what matters most is finding a form of movement that brings joy and satisfaction.

## 5 Conclusion

In this study, Norwegian adolescents participating in XC skiing revealed the highest satisfaction in school, highest PA levels, lowest pain levels and lowest use of painkillers. Participation in sports, regardless of sport category, is associated with increased LS of Norwegian adolescents, with the strongest associations unveiled among girls. With this research, we add further insights into adolescents’ life satisfaction, training, pain and painkiller use, providing novel sport-specific knowledge of both healthy and risk-behavior. This study underscores that adolescence is a particularly critical time-window for promoting sports engagement and PA, especially among girls.

## Data Availability

Data supporting the results of this study is available upon request from the Norwegian Agency for Shared Services in Education and Research (SIKT) and NOVA. Reference to dataset from SIKT: (https://doi.org/10.18712/NSD-NSD3157-V1). Request to access these datasets should be directed to https://sikt.no and https://www.ungdata.no/den-nasjonale-databasen/. Further inquiries can be directed to the corresponding author.
